# A Factorial Survey Investigating the Effect of Disclosing Parental Intellectual Disability on Risk Assessments by Children’s Social Workers in Child Safeguarding Scenarios

**DOI:** 10.1093/bjsw/bcz076

**Published:** 2019-06-30

**Authors:** Ameeta Retzer, Jane Kaye, Ron Gray

**Affiliations:** 1 National Perinatal Epidemiology Unit (NPEU), Nuffield Department of Population Health, University of Oxford, Oxford, OX3 7LF, UK; 2 Centre for Health, Law and Emerging Technologies (HeLEX), Nuffield Department of Population Health, University of Oxford, Oxford, OX3 7LF, UK

**Keywords:** children’s social workers, England, factorial survey, parental intellectual disability, risk assessment

## Abstract

Literature suggests that, as parents, people with intellectual disabilities experience disproportionately high rates of child removal compared to other groups. A factorial survey of 191 children’s social workers investigated the effect of disclosing parental intellectual disability (ID) upon risk assessments in a range of hypothetical child safeguarding scenarios. The case scenarios depicted a range of child safeguarding situations and parents’ ID status was randomly included as an additional item of information. The data were fitted into a generalised ordinal logistic regression model. Findings indicate that when presented with scenarios considered to be less risky, the parental ID disclosure contributed significantly to a higher risk assessment score. However, when presented with scenarios that were considered more risky, the additional parental ID disclosure did not significantly contribute to a higher score. These findings indicate that the risk associated with parental ID is not fixed but relative to the situation in which it is encountered. The research concludes that in cases of low risk, the effect of parental ID is identified as a support need, whereas the lesser contribution of the disclosure to assessments of higher risk cases may indicate that parental ID is overlooked.

## Introduction

Decision-making by children’s social workers (CSWs) is affected by a wide range of factors, including their own individual heuristics, their personal values, the agency and legislative context, and characteristics of those with whom they have contact (Drury-Hudson, 1999). CSWs are the professionals that work with children and their families to ensure children’s well-being by continuously monitoring the risk presented to the children in question and acting accordingly. When children are identified to be at risk, this can result in their being placed temporarily or permanently outside of their parents’ care.

Intellectual disability (ID), also referred to as ‘learning disability’ and ‘learning difficulty’ (Holland, 2011), is used to indicate ‘the presence of a significantly reduced ability to understand new or complex information, in learning new skills (impaired intelligence), with a reduced ability to cope independently (impaired social functioning), which started before adulthood, with a lasting effect on development’ (Emerson and Heslop, 2010, p. 1).

Current literature suggests that parents with ID (PWID) are over-represented in terms of the parents whose children are subject to child protection procedures and ultimately removed from their parents’ care. As such, PWID are considered to be at risk of having their children removed. It is not known how many PWID are living in England but people with ID represent approximately 2 per cent of the general population (Holland, 2011). Despite this, of the parents about whom children’s services have concerns, 12.8 per cent of the mothers and 6.8 per cent of fathers are known to have ‘learning difficulties’ (Masson *et al.*, 2008). Studies examining child protection procedure (Booth and Booth, 1996; McConnell and Llewellyn, 2000; Wates, 2002; Llewellyn *et al.*, 2003; Booth *et al.*, 2005) and the experiences of PWID (Booth and Booth, 2004; Baum and Burns, 2007; Gould and Dodd, 2014) suggest case outcomes and parents’ negative experiences may be due to discrimination by practitioners. Fear of discrimination has been cited as why PWID are wary of engaging with parental support services (Gould and Dodd, 2014).

An investigation of social services and courts in England management of child protection cases involving PWID (Booth *et al.*, 2005) found that children of PWID were more likely to be subject to freeing orders than children of other parents. These children were also more likely to be placed out-of-home and outside of their kinship network. The conclusions drawn from such research often describe harsh treatment of parents on the basis of their disability: ‘the problems giving rise to the professionals’ concern and leading them to feel that the situation as such was irredeemable were directly related to … intellectual disability’ (Booth *et al.*, 2005). Unexamined prejudices against those with any form of disability have been cited as cause for the over-representation of children of people with disabilities in the looked-after system (Wates, 2002). These are sentiments that are echoed throughout the international literature (Hayman, 1990; Taylor *et al.*, 1991; Watkins, 1995; Levesque, 1996; Keyzer *et al.*, 1997; Bray, 1999; McConnell and Llewellyn, 2000, 2002; Swain *et al.*, 2003; Proctor and Azar, 2013).

However, two studies conducted in England have examined social services practitioners working with PWID and their views. The first (Cleaver and Nicholson, 2007, 2008) explored assessment, parental involvement, and children’s outcomes and found that social workers worked in partnership with PWID. Social workers felt ID was an obstacle to parent’s involvement and children were placed away from home after substantial service input failed to bring about the required change. There was no evidence parental ID in itself was the reason children were removed. The welfare of a significant proportion of the children in the sample was not being promoted and they were continuing to live in unacceptable situations. The second (Jones, 2013) found ‘an underlying perception that parents with learning difficulties [ID] have limited capacity to change’ and suggested that this could negatively impact upon the assessment process and outcomes. PWID are wary of engaging with parental support services due to fear of discrimination (Gould and Dodd, 2014), but perceived parental ‘non-cooperation’ has been found to be a predictor for the removal of children (McConnell *et al.*, 2011).

This canon of research demonstrates the ambiguities in social work with PWID. The effect of disclosing PID to CSWs while they are making child safeguarding assessments is unclear. This is investigated in this study using factorial survey design, building upon the methods used by Proctor and Azar (2013). We hypothesised that CSWs are more likely to assess children in given child safeguarding scenarios to be at greater risk if parent’s suspected ID status is disclosed than if it was not disclosed.

## Methods

### Sampling of children’s services

Purposive sampling was used to select the twenty-three services, guided by the Children and Family Court Advisory and Support Service (CAFCASS) Care Demand Review (CAFCASS, 2013). CAFCASS Care Demand data were used to select two local authorities from each of the ten English regions, those with the highest and lowest rates of Children in Need (CIN) per 10,000 children, to capture a range of geographies and care burden experienced by participating services (compared to the national average of 343.4). A further three local authorities were approached due to their large population size and socio-economic and cultural diversity. This took place between April and July 2014 and was agreed through liaison with the managerial teams of each children’s service, composed of senior social workers and social work managers. These individuals would contact the CSWs with whom they worked on behalf of the study, to invite them to complete the survey. CSWs were not contacted directly by the researcher due to data protection restrictions.

### Recruitment of survey participants

An invitation to participate in the research and the link to the online survey software were sent by email to the CSWs employed in each service, by their respective managerial teams, and then subsequently in monthly reminders. Participation was voluntary and anonymous and as such, non-participation was permitted without explanation. The frequency of these reminders was adjusted according to the participation rate observed in each children’s service, indicated by the number of registrations to the survey software from each region. In this way, additional efforts were made when recruitment was low or to compensate for poor recruitment in other regions.

### Survey design

This study builds upon the methods in an earlier study of child protection service worker decision-making (Proctor and Azar, 2013). Qualtrics software was used to develop and host the factorial survey. Participants were asked to complete an online factorial survey in which they were presented with nine fictional child safeguarding vignettes to assess. In factorial surveys, the vignettes are systematically constructed using factors thought to be relevant to individuals’ judgement process. Participants are asked to rate the vignettes according to a specified scale. The factors contained within the vignettes are manipulated during the survey while participants continue using the same assessment scale. As a result, any effect of varying the factor can be identified by examining the resulting assessments by the participants.

The term, ‘assessment’, is used in this study to refer to a CSW’s assessment of families’ or parents’ support needs and the level of intervention and urgency that is required from social care actions. Participants indicated their assessments by choosing one of four assessment options that they felt best described their professional view of each case. A modified Likert scale was used to present participants with assessment options that indicated levels of urgency in terms of child protection action: ‘no risk’, ‘early help’, ‘in need’ or ‘significant harm’.

The vignettes included in the factorial survey were composed of two factors of interest—whether Parental ID (PID) had been disclosed and the details of the child protection case scenario. A wide range of child safeguarding scenarios were tested to reflect the types of family situations ordinarily encountered by CSWs. The risk assessment criteria used by the CAFCASS (CAFCASS, 2012) provided the basis for nine safeguarding scenarios depicting a seven-year old child living with his parents, both of whom were suspected to have ID, in situations that may present risk to the child.

Each participant was shown nine vignettes and gave nine assessments, resulting in nine observations from each person. Two versions of the survey were made, each containing the same nine scenarios—one version included the disclosure in four vignettes, the other version including the disclosure in the remaining five. The disclosure of suspected ID was included as an additional item in the final vignette:

(Seven − year − old child living with both parents) ± (disclosure)  + (scenario) Participants were randomly presented with one of the two versions (Table 1). The random inclusion of the PID disclosure in half of the vignettes presented to participants created two experimental conditions, one where PID had been disclosed and the other where it had not been.

**Table 1:** The two versions of the factorial survey vignettes

Scenario [and label]	Version 1	Version 2
Two windows in the room where the child plays are broken and the glass has jagged edges. The child has cut his hand, requiring three stitches [‘glass’]	ID disclosed	ID not disclosed
The parents have been arguing a lot for the past few months and have been quite distracted. The child has not been getting help with his homework from his parents and often has to prepare his own meals [‘argue’]	ID disclosed	ID not disclosed
The child has met some older children who are known to be part of a violent gang. The child is spending less time with his old friends and more time with these older children [‘gang’]	ID disclosed	ID not disclosed
The parents are known to have spoken about the child getting married when he is older, to someone they choose and the child may not know. If such a marriage were proposed, the child may not have a choice [‘marriage’]	ID disclosed	ID not disclosed
The parents have recently adopted a dog from an animal rescue and the child often plays with the dog unsupervised [‘dog’]	ID not disclosed	ID disclosed
The child has been in a number of physical fights with his sister. Most recently, the child has a cut on his lip and a bruise on his head. The child has been injured as a result of these fights in the past [‘sister’]	ID not disclosed	ID disclosed
The child’s mother has self-harmed in the past and is now saying she is feeling depressed and suicidal. The child is aware that his mother feels this way [‘depression’]	ID not disclosed	ID disclosed
The child’s father has recently lost his job and the child’s mother does not work. There is a possibility that the family will not be able to pay their rent as the father’s employment was the main source of income [‘unemployed’]	ID not disclosed	ID disclosed
The child’s maternal uncle has committed an offence that has meant he is now designated a ‘risk to children’. This uncle has regular and sometimes unsupervised contact with the child [‘uncle’]	ID not disclosed	ID disclosed

Participants each had an equal chance of being directed to either set of vignettes, reducing selection bias (Altman, 1991) and the order in which the vignettes were presented was randomised to alleviate any bias in this regard (Krosnick, 1999).

Cognitive interviewing techniques were used to improve the validity and reliability of the questionnaire (Willis *et al.*, 1991; Desimone and Le Floch, 2004), whereby each of the items included in the questionnaire was completed by social worker colleagues, in turn, in the presence of the researcher. These individuals described aloud how they interpreted and understood each item, and the researcher used their feedback to refine the questions to ensure their meanings were clear and would meet their intended purpose.

### Ethical and organisational permissions

This study received ethical clearance from the Medical Sciences Inter Divisional Research Ethics Committee at University of Oxford (Ref: MSD-IDREC-C1-2014–024). A detailed information sheet and consent form were included at the start of the survey. Participants could only access the main body of the survey if they agreed to a consent statement. An application was made to the Association of Directors of Children’ Services (Ref: RGE140128) to invite twenty-three children’s services to take part in this research. This research was undertaken as part of a programme of doctoral research by Ameeta Retzer.

## Analysis

Stata 13 was used to analyse the data. The study collected ordinal, non-dichotomous data. The four assessment items—‘no risk’, ‘early help’, ‘in need’ and ‘significant harm’—indicated levels of severity. These are necessary to ensure that the survey is representative of the spectrum of choices actually available to CSWs, capturing the nuances of their assessments better than would a binary option. The aim of the analysis was to find the extent to which the explanatory variables (the scenario and PID disclosure) would predict the outcome variable, the assessment. Participants’ assessment choices were analysed using generalised ordinal logistic regression to identify the unique net contribution of the factor of interest (Williams, 2006; Fullerton, 2009), the PID disclosure, upon the assessment choice. A model was constructed to contrast the impact of the disclosure of assessment choice compared to where there was no PID disclosure. The disclosure variable was binary, coded 0 when PID was not disclosed and 1 when it had been.

A generalised ordinal logistic model was used whereby the parallel lines assumption was completely relaxed for all of the variables (Williams, 2006). The parallel lines assumption, also referred to as the proportional odds assumption, assumes that the relationship between each pair of outcome groups is the same and that the coefficients describing all of these relationship combinations are the same, resulting in one model and one set of coefficients (UCLA, 2015). This means the effects of the explanatory variables are proportional across the different thresholds of the outcome variable. Where this assumption is violated and a different model is required to describe the relationship between each pair of outcome groups, an approach that relaxes this assumption is needed instead.

Overlooking a violation of the parallel lines assumption would result in biased estimates and the effects of particular independent variables being obscured. In such a model, the positive impact of a variable at one level may be countered by its opposite effect at another, resulting in a non-significant effect. In an alternative model where the parallel lines assumption is relaxed, there would be two separate significant effects. Use of such an alternative model reduces bias in the coefficients and uncovers more nuanced relationships (Fullerton, 2009).

To test whether the parallel lines assumption was violated, a likelihood ratio test was performed. A non-significant result would indicate that there is no difference in the coefficients between the models, indicating that the assumption had not been violated (UCLA, 2015). However, an approximate likelihood ratio test of proportionality of odds across the response categories gave a significant result (*p* > *χ*^2^ = 0.0050), indicating a definite violation of the assumption for the independent variable under study—the disclosure.

## Results

### Explanatory variables

Due to the survey design, the ID and non-ID assessments were taken from the same participants and all participants assessed vignettes containing the same nine scenarios so there were no significant differences between the ID and non-ID groups. When grouped by region, no significant difference was identified. For these reasons, the PID disclosure alone was included in the final model. This resulted in 1,719 assessments.

### Sample characteristics

Recruitment resulted in the participation of six children’s services. The final study sample amounted to 20.6 per cent (*n* = 191) of the total study population of 925 (Table 2), with great variation across regions (Table 3). Children’s services and CSWs were not required to give reasons for their non-participation. However, two of the services declined to participate due to workload pressures upon their staff.

**Table 2:** Participant characteristics

Gender	Number of participants
Male	21
Female	167
Prefer not to say	3
Age	
20–29	25
30–39	59
40–49	53
50–59	44
≥60	8
Prefer not to say	2
Ethnicity	
White	178
Mixed race	4
Asian/Asian British	1
Black/Black British	3
Arab/Other	2
Prefer not to say	3

**Table 3:** Sample characteristics

Children’s service	Final number of participants per service in sample (*N* = 191), *n* (%)	Percentage recruited from service (%)	Rate of CIN per 10,000 children and regional average
1 Northeast	43 (22.51)	35.83	325.0 (443.8)
2 Northeast	32 (16.75)	45.71	734.9 (443.8)
3 Southwest	3 (1.57)	3.00	171.3 (320.0)
4 Southwest	53 (27.75)	17.67	211.6 (295.7)
5 Yorkshire and Humber	12 (6.28)	6.00	542.5 (346.3)
6 West Midlands	47 (24.61)	32.41	528.3 (343.7)
Prefer not to say	1 (0.52)		

In generalised ordinal logistic regressions, positive coefficients indicate that higher values of the explanatory variable (the disclosure variable) make it more likely that the participant will be in a higher category of the outcome variable (the assessment) than the current one. Negative coefficients indicate the opposite that a higher value of the explanatory variable will increase the likelihood of being in the current or lower category (Williams, 2006). The coefficients in this model indicate that the assessment was likely to be in a higher category than ‘no risk’ when PID was disclosed (Table 4). This effect is the same for the other categories that when PID was disclosed, the assessment was likely to be in a higher category than ‘early help’ and ‘in need’. However, the model showed that while this relationship was highly significant in the lower categories, the significance of the PID disclosure decreased as the assessment category increased.

**Table 4:** Generalised ordinal logistical regression coefficients

Assessment	Coefficient	*Z*	*p* > [*z*]	95% confidence interval
No risk	0.590	4.23	0.000	0.316, 0.863
Early help	0.145	1.95	0.051	−0.000, 0.291
In need	0.115	1.47	0.142	−0.038, 0.926

### Interpretation

The aim of the factorial survey was to isolate the effect of an ID disclosure upon CSWs assessments of a child safeguarding scenario by manipulating ID disclosure in the vignettes. The effect of varying this factor can be identified by examining the resulting assessments by the participants (Table 5).

**Table 5:** Categories of assessment when ID is disclosed or undisclosed

Assessment	ID undisclosed (*N* = 862)	ID disclosed (*N* = 857)
No risk	135	80
Early help	263	285
In need	243	253
Significant harm	221	239

These results indicate that there is small but significant association between PID disclosure and CSW assessments. The nuances of this relationship were uncovered by the generalised ordinal logistic regression wherein the effect of the PID disclosure was most likely to increase the risk assessment from a lower category to a higher one but had lesser impact where the assessment was already one indicating high risk, for example, a scenario that is assessed to indicate a child is ‘in need’ or potentially facing ‘significant harm’ without the additional ID disclosure.

These changes in assessment indicate that the CSWs, when presented with a case and told that the parents in question have ID, were more likely to consider the family to have higher support needs than those where ID was not mentioned. However, this was only the case when the families were depicted in a scenario that, when presented without the ID disclosure, was assessed to be low risk by the participants. In these situations, when PID was mentioned, it contributed significantly to an assessment of higher risk. When families were depicted in a scenario that was assessed by the participants to be high risk with no mention of PID, the additional disclosure of PID did not significantly contribute to the higher assessment of risk.

This indicates that PID affects assessments in low-risk scenarios, such as those that elicit a ‘no risk’ or ‘early help’ assessment. When there are other more substantial signifiers of potential harm to a child, such as those assessed to be ‘in need’ or in ‘significant harm’, PID affects the assessment less. Table 6 illustrates the spread of the assessments across the categories according to the vignettes displayed.

**Table 6:** Distribution of assessments across categories according to vignette

Vignette	Assessment			
‘Unemployed’	No risk	Early help	In need	Significant harm
PID disclosed (*N* = 93), % (*n*)	11.8 (11)	73.1 (68)	15.1 (14)	0 (0)
PID undisclosed (*N* = 98), % (*n*)	35.7 (35)	53.1 (52)	11.2 (11)	0 (0)
‘Uncle’	No risk	Early help	In need	Significant harm
PID disclosed (*N* = 93), % (*n*)	0 (0)	2.1 (2)	8.6 (8)	89.2 (83)
PID undisclosed (*N* = 98), % (*n*)	0 (0)	1.0 (1)	9.2 (9)	89.8 (88)
‘Sister’	No risk	Early help	In need	Significant harm
PID disclosed (*N* = 93), % (*n*)	1.0 (1)	25.8 (24)	29.0 (27)	44.1 (41)
PID undisclosed (*N* = 98), % (*n*)	2.4 (2)	46.9 (46)	30.6 (30)	20.4 (20)
‘Marriage’	No risk	Early help	In need	Significant harm
PID disclosed (*N* = 98) , % (*n*)	18.4 (18)	40.8 (40)	23.5 (23)	17.3 (17)
PID undisclosed (*N* = 93) , % (*n*)	25.8 (24)	25.8 (24)	30.1 (28)	18.3 (17)
‘Glass’	No risk	Early help	In need	Significant harm
PID disclosed (*N* = 98) , % (*n*)	0 (0)	18.4 (18)	39.8 (39)	41.8 (41)
PID undisclosed (*N* = 93) , % (*n*)	4.3 (4)	24.7 (23)	31.2 (29)	39.8 (37)
‘Gang’	No risk	Early help	In need	Significant harm
PID disclosed (*N* = 98) , % (*n*)	1.0 (1)	39.8 (39)	38.8 (38)	20.4 (20)
PID undisclosed (*N* = 93) , % (*n*)	3.2 (3)	36.6 (34)	31.2 (29)	29.0 (27)
‘Dog’	No risk	Early help	In need	Significant harm
PID disclosed (*N* = 93) , % (*n*)	52.7 (49)	35.5 (33)	8.6 (8)	3.2 (3)
PID undisclosed (*N*=98) , % (*n*)	68.4 (67)	27.6 (27)	4.1 (4)	0 (0)
‘Depression’	No risk	Early help	In need	Significant harm
PID disclosed (*N* = 93) , % (*n*)	0 (0)	20.4 (19)	51.6 (48)	28.0 (26)
PID undisclosed (*N* = 98) , % (*n*)	0 (0)	23.5 (23)	52.0 (51)	24.5 (24)
‘Argue’	No risk	Early help	In need	Significant harm
PID disclosed (*N* = 98) , % (*n*)	0 (0)	42.9 (42)	49.0 (48)	8.2 (8)
PID undisclosed (*N* = 93) , % (*n*)	0 (0)	35.5 (33)	55.9 (52)	8.6 (8)

As such, it can be concluded that PID has a significant effect on assessments of cases that would otherwise not be considered to be of great concern, and an insignificant effect on assessments of cases that, when presented alone, would be considered highly risky. This effect is demonstrated in Table 6 by the ‘uncle’ and ‘unemployed’ scenarios. In the case of ‘uncle’, where the hypothetical seven-year-old child is left unsupervised with an uncle deemed to be a ‘risk to children’, that scenario alone was considered enough to warrant the participants choosing a ‘significant harm’ assessment. The PID disclosure had limited additional impact. However, in the case of ‘unemployed’, where the father of the seven-year-old child has lost his job and the family may be unable to pay their rent, the effect of the PID on the lower level assessments is evident. More participants assessed the family to require ‘early help’ or as ‘in need’, and were less likely to choose ‘no risk’ than if PID was not disclosed.

## Discussion

Analysis of the factorial survey data showed that PID disclosure had an impact on CSWs’ assessments. CSWs were more likely to choose assessment categories indicative of higher support needs and greater urgency when PID was disclosed. This effect was clearest at the lower end of the assessment categories, where the disclosure escalated the participants’ assessments. In the higher categories, the contribution of the PID disclosure to the assessment was not found to be significant—the degree to which the disclosure made an impact was unclear.

These findings indicate that PID is considered by CSWs as a factor amongst the other defining characteristics of a case. Its relevance is only determined relative to the other factors at play. This might mean that in the absence of other starkly worrying factors, PID would be subject to more investigation. This is not necessarily an indicator of negative or discriminative assessments, but a demonstration that CSWs are aware these parents may have additional support needs that would need clarifying. In cases where there are other factors that alone would result in a ‘significant harm’ or ‘in need’ assessment, it is unclear how the PID disclosure is included in the assessment.

Further research is necessary so that the reasoning for these decisions may be explored. There was no means within the survey instrument for participants to elaborate on how they made their assessments. The addition of a qualitative item that would allow participants the opportunity to explain their choice of assessment would have provided more insight. Such an item would have contributed to the length of the questionnaire and the time required for its completion. These may have had a detrimental effect on participation and the added length would have made the study less attractive to children’s services during recruitment. As such, a separate study of this is required. The survey in its final form resulted in a relatively low response rate of 20.6 per cent, compared with other social worker surveys (Skills for Care, 2015; Local Government Association, 2018), which may undermine its representativeness. There were substantial variations in response rate between regions. The differences in response rate demonstrate the varying success of the recruitment methods used. The children’s services were chosen for their diverse child protection profiles; however, this would not have been reflected in the final sample.

A strength of this study is that it has isolated PID as a factor in the decision-making of CSWs and identified its effect upon the resulting risk assessment and has done so in a range of scenarios applicable to the realities of the situations encountered by CSWs and the assessment options reflecting those that would normally be available.

Rather than providing numerical Likert scales for participants to give their responses to the vignettes, a modified Likert scale was used. Numerical Likert scales are less informative of participants’ answers than vignette-based subjective threshold scales because they require interpretation by the researcher to fathom what the participants meant in choosing a certain number (Heine *et al.*, 2002; Van Soest *et al.*, 2007). Instead, marking the intervals on a Likert scale with mini vignettes that each outlined a specific response rather than asking participants to rate their response numerically is more unequivocal and helpful for drawing meaning from participants’ answers (Van Soest *et al.*, 2007). In this research, participants were given a range of response options in the form of mini vignettes that detailed a specific child protection action. In practice, CSW assessments vary according to the severity of the risk presented to a child and there is guidance on appropriate action to be taken in specific circumstances (DfE, 2013). Using the ‘no intervention’, ‘early help’, ‘child in need’ and ‘significant harm’ options removed the ambiguity about what participants meant and participants also were familiar with and could be guided by the options presented. As a result, it may be surmised that the evidence gathered in this study is entrenched in the realities of CSWs in the England and reflect their practice.

This research has found that rather than a widely held negative view of parents with ID, the defining factor that determines the assessment of cases appears to be the other factors that are present. The perceived risk assessed to be presented by these factors affects the extent to which PID contributes to the overall risk assessment. Where PID presents along with high risk factors, it does not affect the risk assessment, whereas where PID presents along with low risk factors, it does affect the risk assessment and results in a greater assessment of risk. Rather than an active form of discrimination as is suggested by the wider literature, this study illustrates a different pattern of assessment. This provides a basis for further research into the specifics of how PID is considered alongside other factors.

There are studies that contradict the view that PWID are negatively regarded by professionals working with children, one of which is study of child protection service worker decision-making in the USA (Proctor and Azar, 2013). This study, upon whose methods the current research builds, used vignettes to investigate the effect PID disclosure had upon workers’ ‘emotional reactions, attributions and decisions about risk to the child, whether to remove the child and workers’ general willingness to help the parent’ (Proctor and Azar, 2013, p. 1104). While they found that perceived increased risk to children was significantly associated with PID disclosure, so was workers’ willingness to help. Also, lower feelings of anger, higher ratings of pity and greater willingness to help were also observed for cases with PID.

In addition to the emotional aspects of their work, CSWs are subject to a range of internal, external and systemic forces during their work with families headed by PWID. The extent to which services systems and the individuals within them are equipped to meet the needs of PWID appears to be subject to great variation. The gap between professionals’ and systems’ capability to meet these needs and the demand and the effect of failing to address this shortfall has been demonstrated (Azar and Read, 2009).

Individuals with ID and children are two groups to whom certain obligations of protection and provision are held. The subject of parenting and ID is highly emotive and the needs of these two groups are often placed on opposing sides. Poor outcomes for children are perceived to be tied to the perceived shortcomings of parents and as such, support for one can be considered to be detrimental to the support required for the other. The field of research also appears to take this shape and a cleft emerges, separating investigation into two ‘sides’—one considering the needs, experiences and interests of parents, and the other catering for those of children. While parents’ experiences of child protection procedure and parenthood are well documented (Booth and Booth, 1995; Aunos *et al.*, 2005; Booth and Booth, 2005; MacIntyre and Stewart, 2012; Mayes and Llewellyn, 2012), this study aimed to bridge the two sides and draw upon the insights and experiences of CSWs to better understand their work with PWID.
